# Dr. David Prince

**Published:** 1890-02

**Authors:** 


DR. DAVID PRINCE, of Jacksonville, Ill., died at his home, December 19, 1889, in the seventy-fourth year of his age. Dr. Prince was a surgeon of high renown, a medical writer of clearness and force, and a physician who enjoyed the confidence of his friends and neighbors, as well as the respect and friendship of his professional brethren, which latter have adopted appropriate memorial expessions thereof.

